# Chromatin Ring Formation at Plant Centromeres

**DOI:** 10.3389/fpls.2016.00028

**Published:** 2016-02-15

**Authors:** Veit Schubert, Alevtina Ruban, Andreas Houben

**Affiliations:** ^1^Leibniz Institute of Plant Genetics and Crop Plant Research (IPK) GaterslebenStadt Seeland, Germany; ^2^Department of Genetics, Biotechnology, Plant Breeding and Seed Science, Russian State Agrarian University - Moscow Timiryazev Agricultural AcademyMoscow, Russia

**Keywords:** CENH3, centromere organization, interphase nucleus, meiosis, mitosis, repetitive DNA, super-resolution microscopy

## Abstract

We observed the formation of chromatin ring structures at centromeres of somatic rye and *Arabidopsis* chromosomes. To test whether this behavior is present also in other plant species and tissues we analyzed *Arabidopsis*, rye, wheat, *Aegilops* and barley centromeres during cell divisions and in interphase nuclei by immunostaining and FISH. Furthermore, structured illumination microscopy (super-resolution) was applied to investigate the ultrastructure of centromere chromatin beyond the classical refraction limit of light. It became obvious, that a ring formation at centromeres may appear during mitosis, meiosis and in interphase nuclei in all species analyzed. However, varying centromere structures, as ring formations or globular organized chromatin fibers, were identified in different tissues of one and the same species. In addition, we found that a chromatin ring formation may also be caused by subtelomeric repeats in barley. Thus, we conclude that the formation of chromatin rings may appear in different plant species and tissues, but that it is not specific for centromere function. Based on our findings we established a model describing the ultrastructure of plant centromeres and discuss it in comparison to previous models proposed for animals and plants.

## Introduction

Centromeres of eukaryotic chromosomes are regions where spindle fibers attach to perform chromatid or homolog separation during mitosis and meiosis. Structurally, different types of centromeres exist (Cuacos et al., 2015). Commonly, they represent a distinct single primary constriction (monocentric chromosomes). But they may also be undiscernible (no primary constriction at very small chromosomes), as e.g., described in *Giardia intestinalis* Kofoid and Christiansen 1915 (Tůmová et al., 2015). Primary constrictions can be elongated to several microns (polycentric chromosomes) as found e.g., in wallaby hybrids (Metcalfe et al., 2007), *Lathyrus* and pea (Neumann et al., 2012, 2016), or form a groove along both sister chromatids of holocentric chromosomes, e.g., of the wood rush *Luzula elegans* LOWE (Heckmann et al., 2011; Wanner et al., 2015).

Centromeres are not conserved at the DNA sequence level and evolutionary long-established centromeres are frequently formed on long arrays of satellite repeat DNAs and/or transposable elements (Henikoff et al., 2001; Jiang et al., 2003; Plohl et al., 2014). In most eukaryotes the histone variant CENH3 serves as a marker for centromeric chromatin and assembles in many species on such specific families of repetitive DNA sequences (Houben and Schubert, 2003; Marques et al., 2015).

Animal centromeres form a trilaminar chromatin-protein complex composed of centromere chromatin and kinetochore proteins (Blower et al., 2002; Sullivan and Karpen, 2004; Ribeiro et al., 2010; Screpanti et al., 2011). Contrary, for meiotic plant centromeres a “ball in a cup” kinetochore configuration was postulated (Bajer and Mole-Bajer, 1972; Dawe et al., 2005). Ultrastructural studies at somatic metaphase centromeres of plants showed that CENH3-containing chromatin forms “curved pad” structures at the surface and sub-surface periphery of the primary constriction where spindle fibers attach (Wanner et al., 2015).

Previously, we identified the formation of ring-like structures at somatic metaphase chromosomes of rye by immunostaining with CENH3-specific antibodies (Banaei-Moghaddam et al., 2012) and in *Arabidopsis thaliana* (L.) Heynh. in interphase nuclei using a centromere-specific repetitive DNA sequence as FISH probe (Schubert et al., 2012). In this study we applied Structured Illumination Microscopy (SIM) which allows the identification of structures beyond the diffraction limit of light at a lateral resolution of ~120 nm and an axial resolution of ~250 nm (Schermelleh et al., 2010), to clarify whether centromeric chromatin ring formation is a common feature at plant monocentromeres. Therefore, we investigated *Arabidopsis* and cereal centromeres during cell division and interphase in different tissues.

## Materials and methods

### Plant material

The following species were analyzed: *Arabidopsis thaliana* (L.) Heynh. (2n = 10), *Secale cereale* L. (rye, 2n = 14), *Triticum aestivum* L. (wheat, 2n = 42), *Aegilops speltoides* ssp. *aucheri* (Boiss.) Chennav. (2n = 14+supernumerary B chromosomes) and *Hordeum vulgare* L. (barley, 2n = 14).

### Slide preparation, immunostaining, and FISH

Flower buds and/or root tips of wheat, rye and barley were fixed for 45 min in ice-cold 4% (w/v) paraformaldehyde in 1xMTSB buffer (50 mM PIPES, 5 mM MgSO4, and 5 mM EGTA, pH 7.2). After washing in 1xMTSB, chromosome spreads were prepared by squashing. Young *Ae. speltoides* spikes were pretreated in ice-cold water for 24 h and then fixed in ethanol:acetic acid (3:1) for at least 4 days. Afterwards, the spikes were stained with aceto-carmine and chromosomes were prepared by squashing in 45% acetic acid.

Tissue sections of *Ae. speltoides* were prepared according to Steedman (1957) and Braszewska-Zalewska et al. (2013). Briefly, developing seeds were excised from spikelets and fixed in freshly prepared 3% (w/v) paraformaldehyde with 0.05% Triton X100 in 1 × PBS buffer on ice for 5 h. Then, dehydration was performed in an ethanol series (ethanol/1 × PBS buffer) from 30 to 96% for 30 min in each at room temperature and in 96% for 30 min at 37°C. Afterwards, the tissues were infiltrated with PEG1500-wax and embedded in a small casting mold (1–3 seeds per block). The blocks were cut into 10 μm slices using a Leica microtome (RM2265; knifes 35N from Feather company). The slices were transferred onto poly-L-lysine-coated slides using forceps and a brush and were stretched by adding a drop of 1 μl water over each slice. The rest of PEG-wax was removed from the dry slides by washing them in 90% ethanol.

Differentiated 2–16C leaf nuclei of *A. thaliana* were isolated and flow sorted according to their DNA content from differentiated rosette leaves after formaldehyde fixation using a FACS Aria (BD Biosciences) as described (Pecinka et al., 2004).

To evaluate the substructure of CENH3 containing chromatin, immunostaining was performed according to Jasencakova et al. (2000). CENH3 was detected with rabbit anti-grass CENH3 primary antibodies (Sanei et al., 2011) and goat anti-rabbit rhodamine (1:300; Jackson Immuno Research Laboratories) or goat anti-rabbit Alexa488 secondary antibodies (1:200; Molecular Probes). Spindle fibers were labeled with monoclonal mouse anti α-tubulin (1:200; clone DM 1A, Sigma) and anti-mouse Alexa488 (1:400; Molecular Probes) antibodies.

For FISH the 180-bp centromeric repeat sequence pAL of *A. thaliana* (Martinez-Zapater et al., 1986) was generated by PCR as described (Kawabe and Nasuda, 2005). The centromeric retrotransposon CRW2 of wheat was generated by PCR as described by Li et al. (2013). These probes as well as the subtelomeric repeat HvT01 (Schubert et al., 1998) and BAC7 containing centromere-specific repeats of barley (Hudakova et al., 2001; Houben et al., 2007) were directly labeled by nick translation with TexasRed-dUTP, Alexa488-dUTP and Cy3-dUTP according to Ward (2002). FISH was performed according to Schubert et al. (2001).

For the colocalization of CENH3 immunosignals with centromeric FISH signals, immunostaining was performed first. The slides were treated with 10 mM citrate buffer (pH 6) in a microwave at 800 Watt for 60 s according to Chelysheva et al. (2010). Then the primary antibodies were applied and immunostaining was performed as described (Jasencakova et al., 2000). Prior FISH the slides were treated with ethanol:acetic acid (3:1) fixative for 10 min and freshly prepared 4% formaldehyde in 1 × PBS for 10 min, followed by three times washing for 5 min in 1 × PBS. These steps are important to stabilize the immunosignals during the following FISH procedure, which was performed as described (Ma et al., 2010). Nuclei and chromosomes were counterstained with DAPI (1 μg/ml) in Vectashield (Vector Laboratories).

### Super-resolution microscopy

To analyse the substructure of chromatin beyond the classical Abbe/Raleigh limit (super-resolution) spatial Structured Illumination Microscopy (3D-SIM) was applied using a C-Apo 63 × /1.2 W Korr objective of an Elyra PS.1 microscope system and the software ZEN (Carl Zeiss GmbH). Images were captured using 405, 488, and 561 nm laser lines for excitation (42, 34, and 28 μm grids for 561, 488, and 405 nm excitations; 5 rotations) and the appropriate emission filters. 3D-SIM stacks with a step size of 110 nm were acquired consecutively for each fluorophore starting with the highest wavelength dye to minimize bleaching. SIM image stacks were used to produce 3D movies by the Imaris 8.0 (Bitplane) and ZEN 2012 software.

## Results

We applied centromere- and subtelomere-specific DNA repeats and antibodies against CENH3 to investigate the ultrastructure of specifically labeled chromatin and to check whether the chromatin ring formation is a specific feature of centromeres in monocot plants and the eudicot species *A. thaliana*.

### Centromere chromatin ring formation appears in arabidopsis nuclei of different endopolyploidy

**Figure 1:**
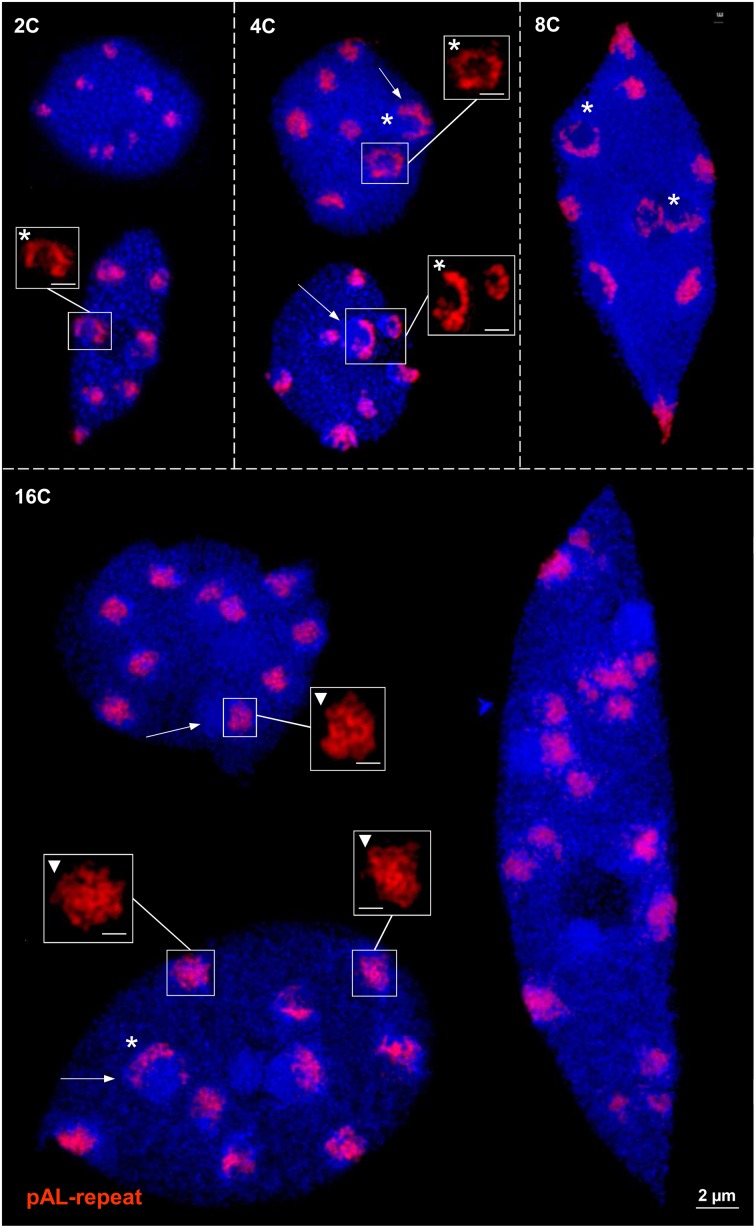
**Centromeric chromatin (pAL) ring formation in differentiated 2–16C leaf nuclei of *A*. *thaliana***. In addition to globular reticulate chromatin structures (triangles), ring and half-ring formation (asterisks) appears at all ploidy levels, especially around chromocenters (arrows). In 16C nuclei due to loss of cohesion centromeres may become separated but they keep their globular structure as demonstrated by the 17 signals in the right nucleus. Bar size in inset = 0.5 μm.

To test whether there is a varying centromeric chromatin ring formation in interphase nuclei of different endopolyploidy, flow sorted 2–16C differentiated leaf nuclei of *A. thaliana* were analyzed using the centromere-specific DNA repeat pAL as FISH probe. In addition to globular structures, also ring and half-ring structures were identified at all ploidy levels. By SIM it became obvious that these centromere structures are composed of a network of chromatin loops (Figure 1). Ring and half-ring formation appears around chromocenters and especially after centromere association, meaning that centromeres tend to fuse in interphase nuclei. In 16C nuclei due to decreased cohesion aligned centromeric chromatid regions start to separate (Schubert et al., 2006). Then, more than 10 centromere signals (corresponding to the chromosome number in *A. thaliana*) may appear. These centromere regions keep the globular substructure, but no further ring formation was observed (Figure 1).

### Centromere chromatin ring formation is present at rye and wheat chromosomes during mitosis and meiosis

**Figure 2:**
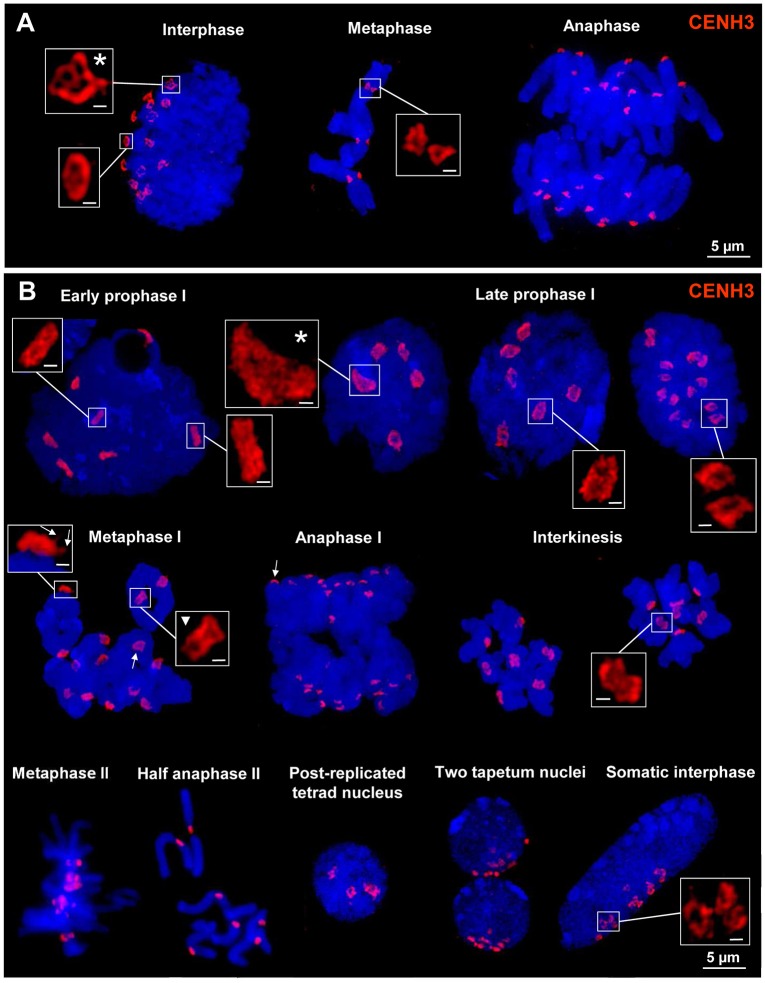
**CENH3 chromatin ring formation at centromeres of rye during mitosis, meiosis and in interphase nuclei. (A)** The 14 CENH3 labeled centromeres visible as rings in interphase become separated at somatic metaphase which segregate at their chromatids during anaphase. The main ring structures may be formed by several subrings (asterisk). **(B)** CENH3 structures during meiosis in pollen mother cells and somatic anther cells. After centromere alignment in early prophase I homologous centromeres coalesce and form ring structures which are split at the end of prophase I. The single rings composed by the two sister centromers present in metaphase I start to separate again (triangle) and in interkinesis clearly two rings are visible at each centromere. They are required to separate the sister chromatids in anaphase II. In prophase I centromeres may also associate (asterisk). At metaphase I and anaphase I bivalents the CENH3 chromatin ring structures are characterized by a cap/crown-like shape comprising extensions where spindle fibers may attach (arrows; see also Supplementary Movies 7–12). Somatic interphase nuclei (two of them in tapetum cells, and single ones in other cells) from anthere tissues show Rabl orientation (Rabl, 1885). Also in these nuclei CENH3 structures may coalesce and form ring structures. Bar size in inset = 0.5 μm.

**Figure 3:**
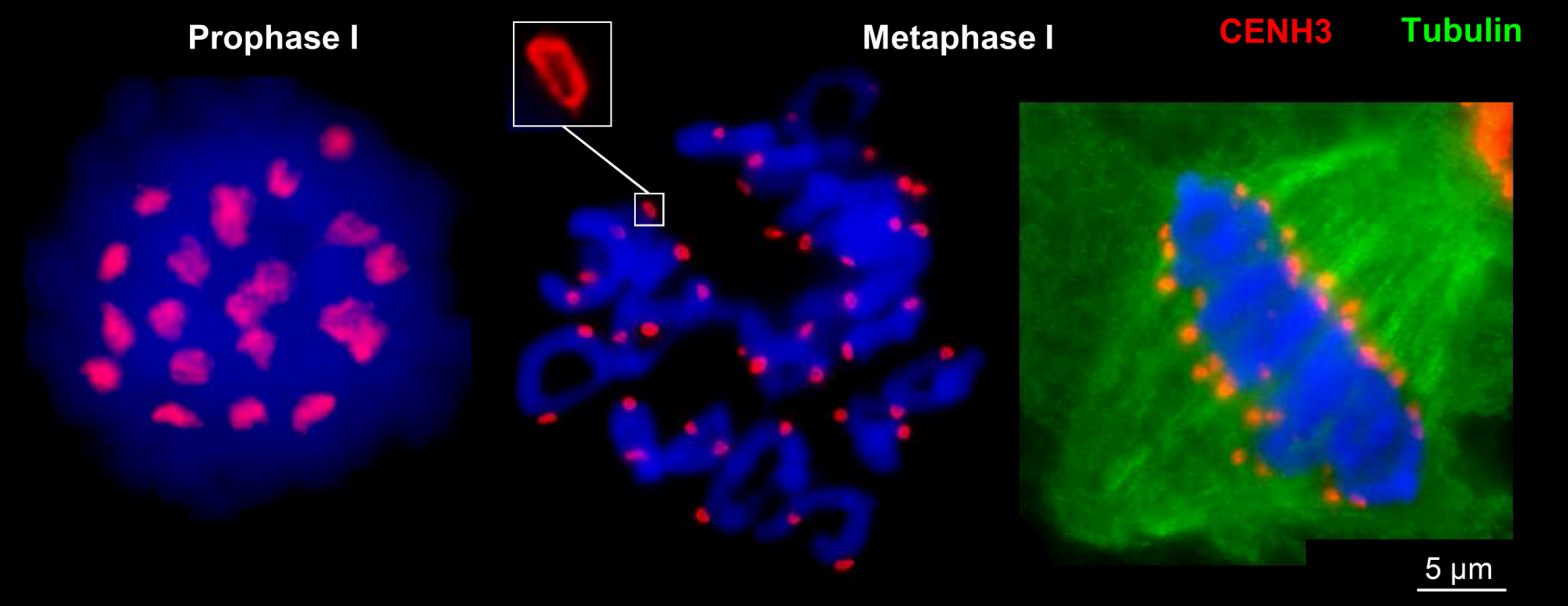
**CENH3-positive centromere structures during meiosis of hexaploid wheat**. In prophase I the four centromeres of paired sister chromatids of both homologs are fused and form ring-like structures which become separated in metaphase I where only two sister chromatids are fused and spindle fibers attach (right).

To investigate the ultrastructure of active centromeres during cell division, rye centromeres were labeled with CENH3-specific antibodies and analyzed by SIM. During mitosis and meiosis as well as in interphase nuclei of roots and anthers CENH3 containing chromatin domains form ring-like structures measuring ~0.5–1.0 μm (Figure 2; Supplementary Movies 1–12). When these rings belonging to single or paired homologs, comprising the centromeres of either two or four sister chromatids, associate or align (e.g., in interphase and prophase I, respectively), they fuse and form bigger rings. During late somatic and meiotic prophase these rings split again and compose smaller ones clearly visible at somatic metaphase chromosomes and at bivalents. Already in metaphase I some of the rings of the sister centromeres split again and become then distantly separated in interkinesis. Similarly, the formation of ring-like structures by CENH3 containing nucleosomes was observed during meiosis of hexaploid wheat (Figure 3).

### Centromere chromatin ring formation varies between aegilops tissues

**Figure 4:**
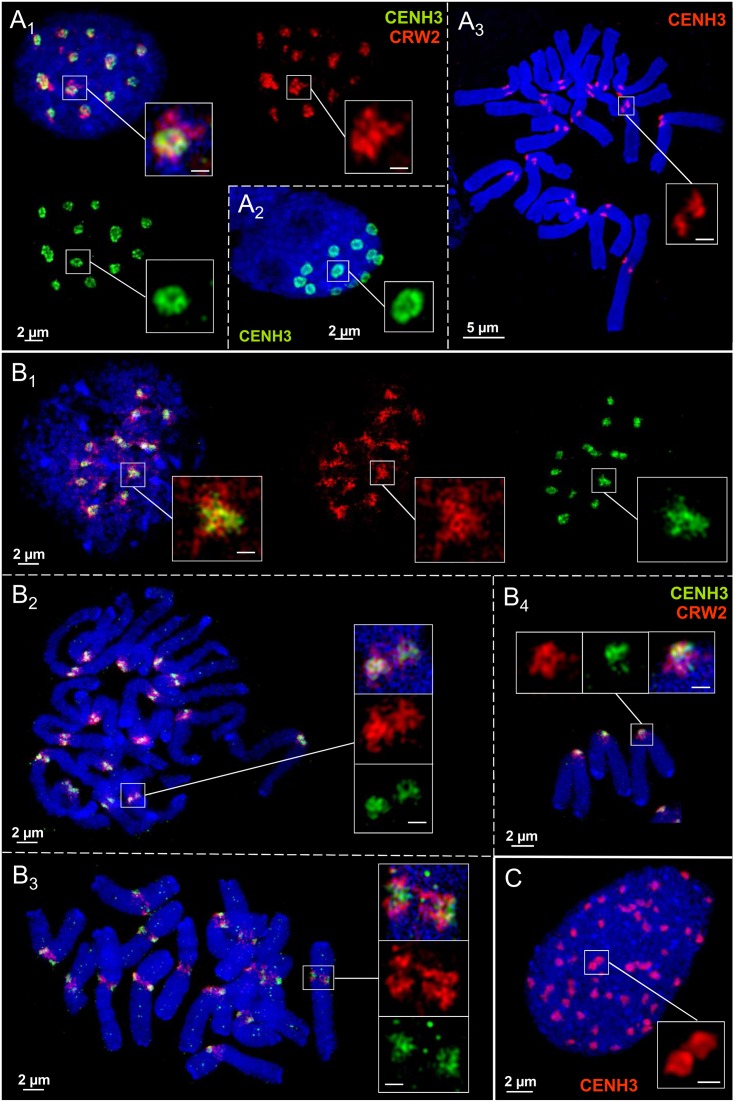
**Centromere chromatin substructures in embryo (A), spike (B), and endosperm (C) tissues of *Ae. speltoides***. CENH3 chromatin ring formation appears pronounced in embryo interphase nuclei **(A**_1−2_**)**, but not at metaphase chromosomes **(A**_3_**)**. The centromeric CRW2 repeat shows mainly a globular organization formed by chromatin fibers present also for CENH3 chromatin in spike tissue during the cell cycle **(B**_1−4_**)**. Due to a high degree of chromatin condensation the CENH3 chromatin ring formation is less clearly visible in endosperm nuclei **(C)**. Bar size in inset = 0.5 μm.

Anti-CENH3 and the centromere-specific repeat CRW2 (Liu et al., 2008) were used to analyse the centromere substructures of *Ae. speltoides*. In most embryonic interphase nuclei the CENH3-labeling shows clearly a ring formation by looped chromatin fibers (Figures 4A_1, 2_) but not at metaphase centromeres (Figure 4A_3_). Irrespective of the presence of supernumerary B chromosomes all chromosomes showed the same centromere chromatin substructure, although we could not identify the Bs based on B-specific probes (Figure 4A_3_; 2*n* = 14 + 2B).

In spike meristems both CENH3 and CRW2 form spherical reticulate substructures during the somatic cell cycle which intermingle among each other. But no ring formation was observed (Figures 4B_1-4_). The differently labeled, but with CENH3 chromatin intermingled CRW2 repeat fibers indicate that most of CRW2 is not associated with CENH3 (Figures 4A_1,_ B_1-4_). The supernumerary chromosomes in pro-metaphase (Figure 4B_2_; 2n = 14+1B) and metaphase (Figure 4B_3_; 2n = 14+2B) obviously do not show a deviating centromere structure.

Compared to embryo nuclei, endosperm nuclei exhibit a more compact CENH3 chromatin organization. Thus, a ring formation is less often visible (Figure 4C).

We conclude that the centromere chromatin organization may differ between tissues of individual *Ae. speltoides* plants.

### Chromatin ring formation appears also outside of barley centromeres

CENH3 antibodies and the centromere-specific repeat containing BAC7 probe of barley (Hudakova et al., 2001; Houben et al., 2007) were applied to label the centromere substructures of barley. For comparison, also the subtelomeric repeat HvT01 (Schubert et al., 1998) was applied as a FISH probe.

**Figure 5:**
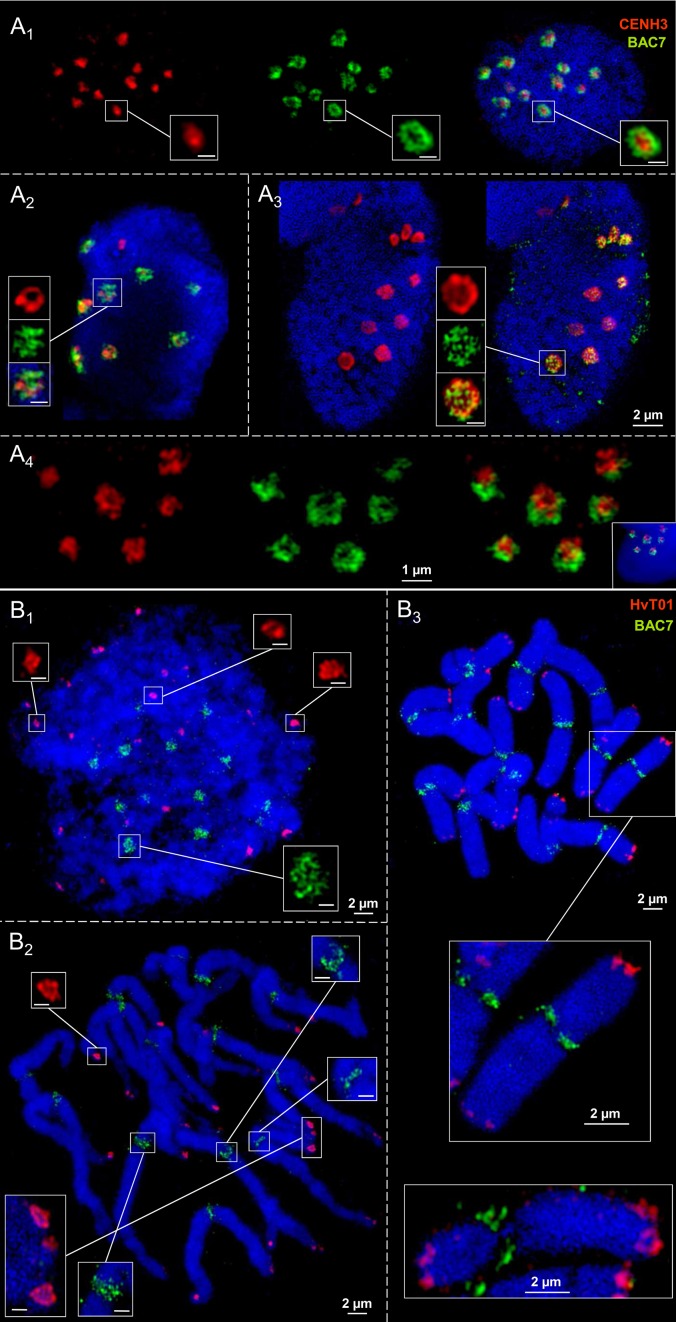
**Ultrastructural organization of centromeres and subtelomeric regions at metaphase chromosomes and in interphase nuclei of barley**. Anti-CENH3 and centromere-specific BAC7-repeats were used as centromeric probes. Repeat HvT01 detects the subtelomeric regions. In interphase nuclei CENH3 chromatin may compose highly condensed globular structures embedded in ring chromatin containing centromeric repeats **(A**_1_**)**. Alternatively, CENH3 chromatin may show ring structures and the centromere repeats (BAC7) may be organized by chromatin fibers in a less condensed globular manner **(A**_2−3_**)**. In addition, both CENH3 and centromeric BAC7 repeats may form rings **(A**_4_**)**. **(B**_1−3_**)** While the centromeric repeats (BAC7) establish reticulate chromatin substructures at centromeres during the somatic cell cycle at centromeres, subtelomeric repeats (HvT01) may form ring-like structures at the subtelomeres in interphase **(B**_1_**)**, prometaphase (**B**_2_**)**, and metaphase **(B**_3_**)**. Bar size in insets = 0.5 μm.

In interphase nuclei both CENH3-positive chromatin and centromeric repeats may establish ring structures (Figures 5A_1-4_). CENH3 chromatin is embedded in centromeric repeats as a condensed globular structure or CENH3-ring-in-centromere repeat-ring configurations may appear. During the somatic cell cycle the centromeric repeats compose reticulate substructure. This is true also for the subtelomeric repeat HvT01, but it may also compose ring chromatin structures (Figures 5B_1-3_). The observation that in identical nuclei differently shaped chromatin substructures occur, excludes that preparation artifacts inducing the ring structure formation arose.

In short, we conclude that the formation of chromatin rings is not a specific feature of centromeres.

## Discussion

Applying super-resolution microscopy we investigated the ultrastructure of centromere chromatin. We found that in different monocot plants and the eudicot species *A. thaliana* centromeric chromatin fibers may establish globular and/or pad-like structures. But also ring structures may appear with varying peculiarities in cycling and differentiated cells depending on the tissue analyzed.

In addition, we demonstrated, although less pronounced, that also chromatin containing subtelomeric repeats may be organized in a ring-like manner. This suggests that repetitive DNA sequences tend to associate as found for *A. thaliana* centromeric (Schubert et al., 2012, 2013) and transgenic repeats along chromosome arms (Pecinka et al., 2005; Jovtchev et al., 2008, 2011; Watanabe et al., 2009). Self-organization and fractal globule formation of chromatin may be models to explain the arrangement of such chromatin segments and its dynamics (Misteli, 2007, 2009; McNally and Mazza, 2010). The observation that non-cycling differentiated endoreduplicated *A. thaliana* nuclei also establish chromatin ring structures at centromeres indicates that this structure is possibly not absolutely required for spindle fiber attachment.

Thus, although there is increasing evidence that an interplay between higher-order chromatin organization and function of nuclei exists (Woodcock and Ghosh, 2010), it remains unclear whether centromeric chromatin rings are essential for kinetochore formation and the attachment of spindle fibers. At least the findings of Bailey et al. (2013) demonstrate that a secondary centromere structure may be important. They showed in human HeLa cells that epigenetic modifications of the N-terminal tail of CENH3 alter the physical properties of chromatin fibers in such a way that the local centromere chromatin organization may be influenced.

**Figure 6:**
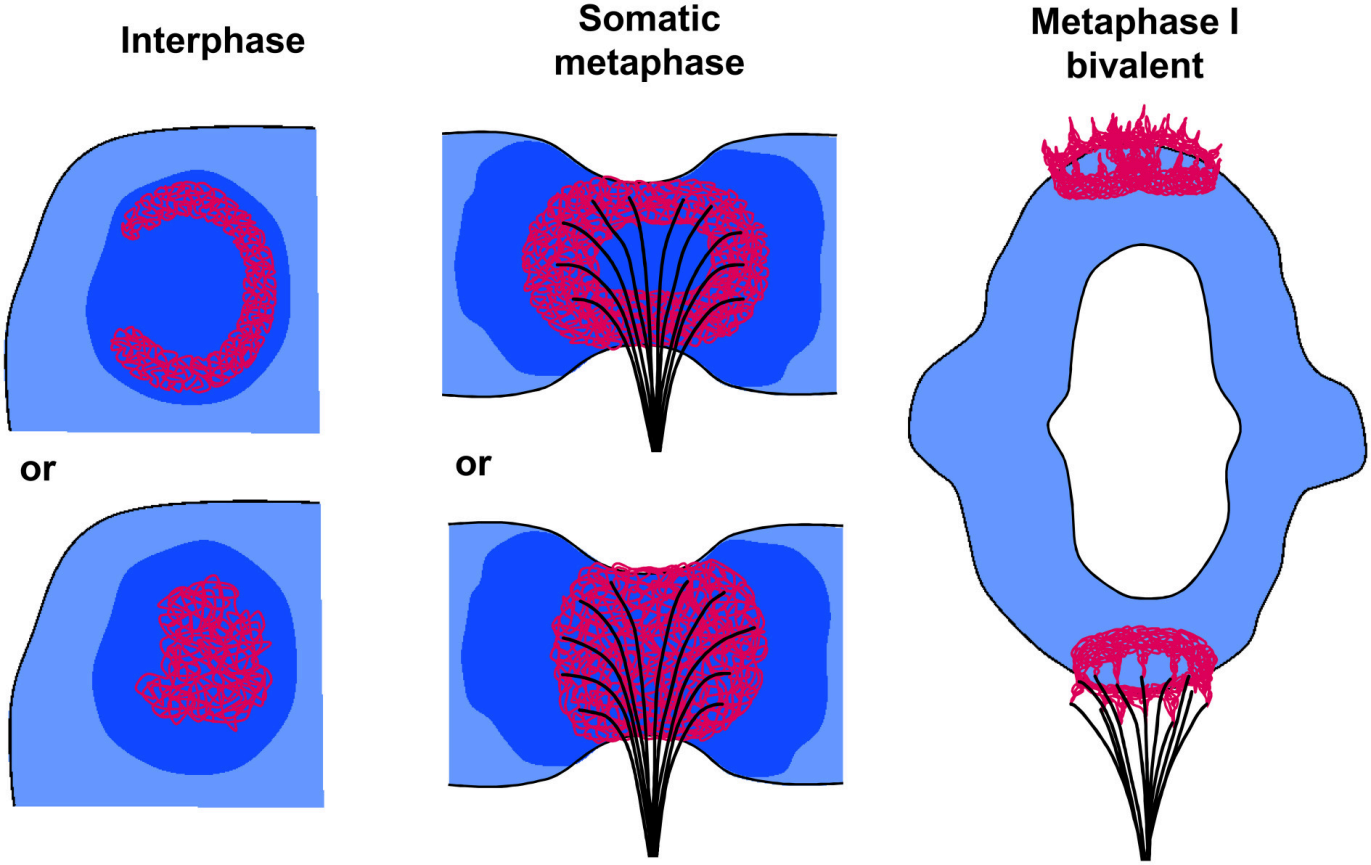
**Models of plant centromere chromatin organization at interphase, somatic metaphase, and metaphase I chromosomes**. In interphase CENH3-containing chromatin (red) may form ring-like (top) or globular (bottom) structures embedded in CENH3-negative heterochromatin (dark blue). At somatic metaphase chromosomes (side view of a single chromatid) the CENH3 chromatin forms rings (top) or globular/pad-like (bottom) structures where spindle fibers attach. The CENH3 chromatin is surrounded by pericentromeric heterochromatin (dark blue). The spindle fibers attach mainly to the pericentromeric flanks of the primary constriction to transfer pulling forces from the microtubules to the more stable chromosome arms (see also Wanner et al., 2015). At metaphase I bivalents, single fused CENH3 chromatin rings composed by the two sister centromers show a crown- like shape (bottom homolog) due to the pulling forces of the microtubules. They become separated again (top homolog) during the transition to anaphase I.

The monocentromeres of barley (Ishii et al., 2015), but also the polycentromeres of *Pisum* and *Lathyrus* (Neumann et al., 2016) have two different CENH3 variants. Both establish globular structures formed by intermingling chromatin fibers, but no centromeric ring formation was observed at somatic metaphase chromosomes. This corresponds to the finding here that the centromeric BAC7 repeats of barley show chromatin fibers only spherically organized at somatic metaphase. The occurrence of pad-like structures at monocentric somatic plant centromeres has also been confirmed by high resolution scanning electron microscopy studies. It became obvious that spindle fibers attach mainly to the pericentromeric flanks of the primary constriction, probably to transfer at least a part of the pulling forces from the microtubules to the more stable chromosome arms to prevent chromosome breakage (Wanner et al., 2015) (Figure 6). This may be one reason why naturally occurring telocentric chromosomes are seldom (Darlington, 1939). However, the telosomes of artificially selected wheat lines containing about half of the CENH3 chromatin amount compared to complete chromosomes are relatively stable (Koo et al., 2015). The prove of Zhang and Dawe (2012) that total centromere size and chromosome size are positively correlated in grass species supports the idea that an adequate amount of CENH3 chromatin is required to maintain the chromosome stability during cell divisions.

Interestingly, scanning electron microscopy studies of meiotic *Tradescantia reflexa* Raf. chromosomes revealed that the centromeres of metaphase I and anaphase I bivalents exhibit a ring-like structure measuring 0.5–0.7 μm in diameter. This structure is localized in a crown-like manner polewards at the bivalents and contains small extensions (Inaga et al., 2000). These observations correspond clearly to shape and size of the centromere ultrastructure we identified by SIM during the meiosis of rye.

Obviously, plant monocentromeres establish a centromere structure deviating from the trilaminar structure of mammals and insects. The ball-in-a-cup organization postulated by Bajer and Mole-Bajer (1972) and adapted by Dawe et al. (2005) for meiotic metaphase I chromosomes of maize may be related to our findings of globular, pad- and/or ring-like CENH3 chromatin structures in such a way that, as we proved here, the CENH3-positive chromatin is mostly centrally embedded in more extended pericentromeric heterochromatin (Figure 6).

In contrast to the B chromosome of rye (Banaei-Moghaddam et al., 2012) we did not find differences regarding the distribution of centromeric repeats between A and B chromosomes of *Ae. speltoides*. However, as we included in our study only one centromeric repeat it remains open whether other centromeric wheat repeats differ between A and B chromosomes in *Ae. speltoides*.

Altogether, we conclude, that centromere ring formation may vary between tissues of one and the same species, between closely related species, but may also be present in more distantly related species. This ring formation may be a matter of repeat self-organization and involved, but not specific for centromere function.

## Author contributions

VS conceived the study and designed the experiments. VS and AR performed the experiments. VS and AH wrote the manuscript. All authors read and approved the final manuscript.

### Conflict of interest statement

The authors declare that the research was conducted in the absence of any commercial or financial relationships that could be construed as a potential conflict of interest. The reviewer ML and handling Editor declared their shared affiliation, and the handling Editor states that the process nevertheless met the standards of a fair and objective review.

## Supplementary material

Supplementary Movie 1 **Somatic oval rye interphase nucleus showing Rabl orientation and ring formation of CENH3 chromatin at centromeres**.

Supplementary Movie 2 **Somatic elongated rye interphase nucleus showing Rabl orientation and ring-like formation of CENH3 chromatin at centromeres**.

Supplementary Movie 3 **Early prophase I of rye showing seven mostly aligned pairs of homologous centromeres**.

Supplementary Movie 4 **Mid prophase I of rye where two pairs of homologous centromeres form already rings**.

Supplementary Movie 5 **Late prophase I of rye where all seven paired homologous centromeres form ring-like structures**.

Supplementary Movie 6 **Late prophase I of rye where one pair of homologous centromeres (center) split into two rings**.

Supplementary Movie 7 **Metaphase I of rye with CENH3 chromatin ring formation at the seven bivalents**.

Supplementary Movie 8 **Metaphase I of rye with CENH3 chromatin ring formation at the seven bivalents**. One pair of homologous centromeres (enlarged) split already into two rings required for spindle fiber attachment in meiosis II.

Supplementary Movie 9 **Metaphase I of rye with a mainly reticulate pad-like CENH3 chromatin arrangement at the seven bivalents**.

Supplementary Movie 10 **Three rye bivalents with CENH3 chromatin showing a cap/crown-like shape induced by spindle fiber tension**.

Supplementary Movie 11 **Rye bivalent with CENH3 chromatin showing a cap/crown-like and reticulate organization**.

Supplementary Movie 12 **Rye bivalent with a dense CENH3 chromatin organization**.
